# Urinary bladder metastasis from suspected second primary breast cancer presenting with ambiguous lower urinary tract symptoms: A case report and literature review

**DOI:** 10.1002/ccr3.7615

**Published:** 2023-06-26

**Authors:** Liao Chang‐Chieh, Ou Yen‐Chuan, Tung Min‐Che, Hu Wei‐Shiang, Tsao Tang‐Yi, Hsu Chao‐Yu

**Affiliations:** ^1^ Department of Medical Education Tungs' Taichung Metro Harbor Hospital Taichung City Taiwan, ROC; ^2^ Division of Urology, Department of Surgery Tungs' Taichung Metro Harbor Hospital Taichung City Taiwan, ROC; ^3^ Department of Anatomical Pathology Tungs' Taichung Metro Harbor Hospital Taichung City Taiwan, ROC

**Keywords:** bladder metastasis, breast cancer, secondary urinary bladder tumors, suspected second primary breast cancer

## Abstract

Common urinary symptoms may arise from metastases from uncommon sites. In patients with a history of cancer, the focus should be on the currently affected organ and the status of the underlying malignancy.

## 1 INTRODUCTION

Globally, breast cancer (BC) is the most common newly diagnosed cancer in females, accounting for 24.5% of all malignancies in 2020.^1^ In the United States, up to 5% of women diagnosed with BC have metastatic disease at the time of first presentation.^2^ Additionally, up to 30% of women with early stage, non‐metastatic BC at diagnosis will develop distant metastases in the future.^2^ Importantly, urinary bladder is an unusual metastatic site for BC, with only 50 cases reported in the last 60 years. The prognosis is usually poor in patients with BC metastasis to the urinary bladder unless it is the only metastatic site.^3^ Here, we present a rare case of a patient diagnosed with urinary bladder metastasis of BC 21 years after the diagnosis of the primary tumor.

## 2 CASE HISTORY AND EXAMINATION

A 67‐year‐old female patient visited the outpatient urology clinic with complaints of increased urinary frequency, urgency, and incontinence; fullness in the lower abdomen; and nocturia. The patient had a history of diabetes mellitus, hypertension, and ureteral stones. She was also diagnosed with right BC 21 years ago. Pathological examination of the tumor biopsy specimen at the time led to the diagnosis of papillary carcinoma, which was positive for estrogen receptor (ER) and progesterone receptor (PR). The cancer stage was T1N0M0 according to the sixth edition of American Joint Committee staging manual. Therefore, she underwent modified radical mastectomy of the right breast. She received tamoxifen therapy for 6 months, was followed regularly in an outpatient setting, and did not receive further treatment owing to the stable disease status. Data on tumor markers, human epidermal growth factor receptor 2 (HER2) status, and other details of the initial diagnosis were not available.

## 3 DIFFERENTIAL DIAGNOSIS

At the current admission, the patient was initially diagnosed with chronic interstitial cystitis. Medical treatment regimens, including solifenacin, tamsulosin, and phenazopyridine were administered for 4 months but did not relieve the symptoms. The patient was advised to undergo intravesical instillation of hyaluronic acid, which she declined.

## 4 INVESTIGATIONS AND TREATMENT

**FIGURE 1:**
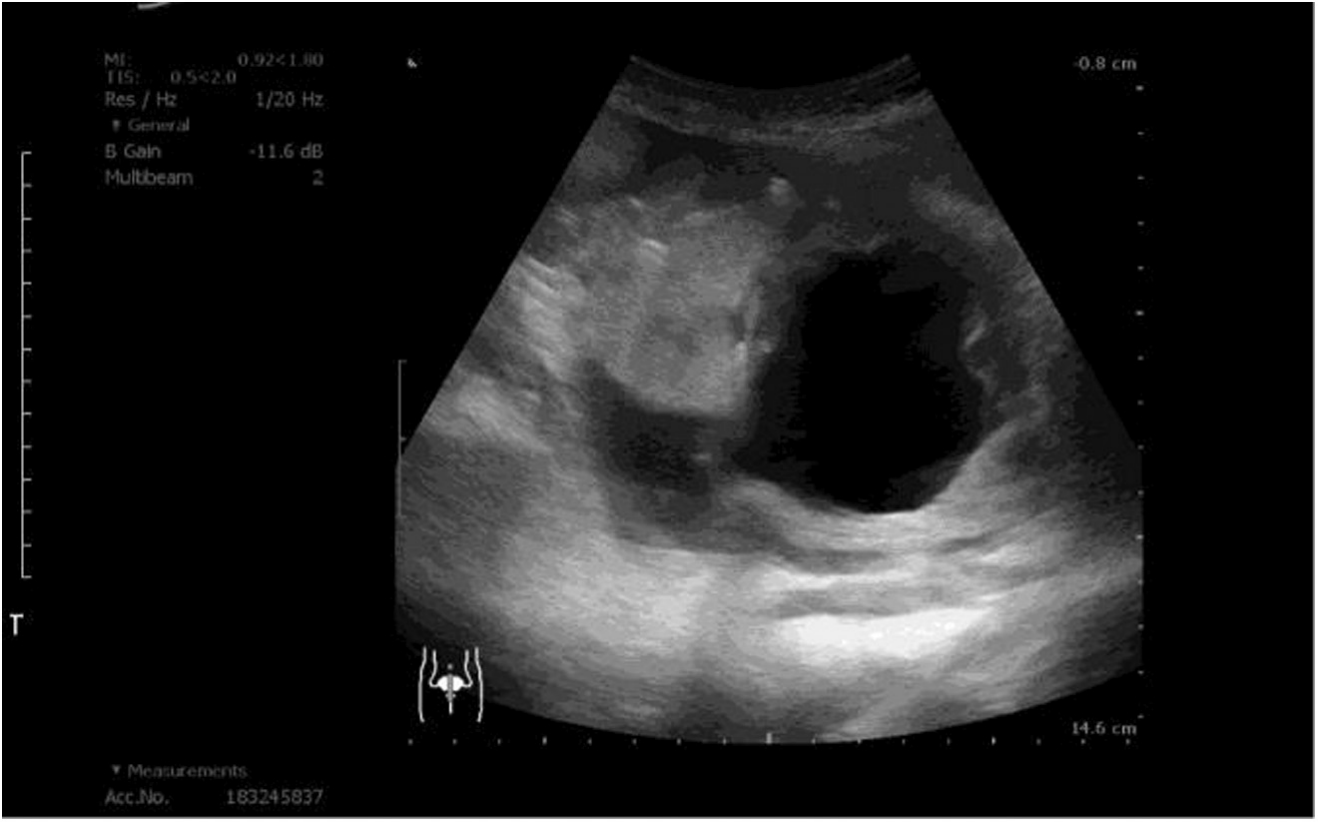
Ultrasound image showing diffuse thickening of the urinary bladder wall that is most notable on the right posterior wall.

**FIGURE 2:**
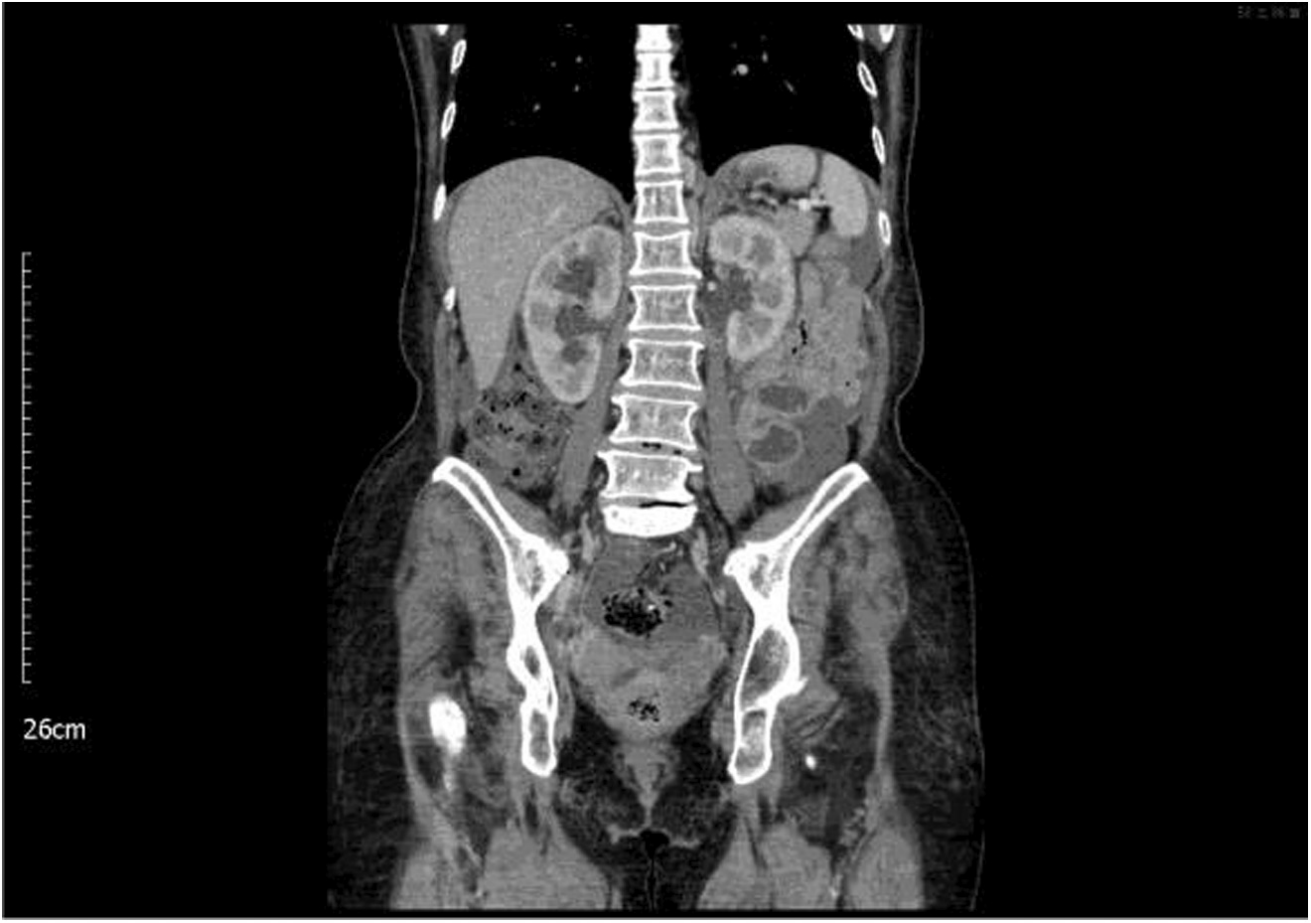
Abdominal computed tomography showing strictures in the lower third of bilateral ureters with bilateral hydronephrosis.

During her first visit of our outpatient department, her uroflowmetry showed poor maximum and mean flow rate, prolonged voiding duration, and a post‐voiding volume of 132 mL. The voiding efficacy was 33%. Routine urinalysis was negative except for + 4 for glucose. However, ultrasound of the urinary bladder revealed a tumor in the right posterior bladder wall and diffuse irregular thickening of the urinary bladder wall (Figure 1). Intravenous pyelography showed bilateral hydronephrosis with poor presentation of filling defects. Chest and abdominal computed tomography (CT) showed lymph node enlargement and strictures in the lower third of bilateral ureters with hydronephrosis (Figure 2). Further testing revealed elevated levels of the following tumor markers: alpha‐fetoprotein, 9.91 ng/mL (normal range, <8.78 ng/mL); carcinoembryonic antigen (CEA), 3536.14 ng/mL (normal range, 0–5 ng/mL); carbohydrate antigen 125 (CA‐125), 2339 U/mL (normal range, <35 U/mL); and carbohydrate antigen 199 (CA‐199), 6673.25 U/mL (normal range, <37 U/mL). The carbohydrate antigen 15–3 (CA15‐3) level was normal (16.3 U/mL; normal range, <31.3 U/mL). Urine cytology examination indicated atypical urothelial cells. Bladder tumor of unknown origin was considered, and cystofibroscopy was performed to collect random biopsy specimens. Inflammatory changes in the urinary bladder observed during cystofibroscopy were compatible with bladder wall thickening. Bilateral endoscopic dilatation of ureter and double‐J stent implantation were performed for treating strictures in the lower third of bilateral ureters with hydronephrosis.

**FIGURE 3:**
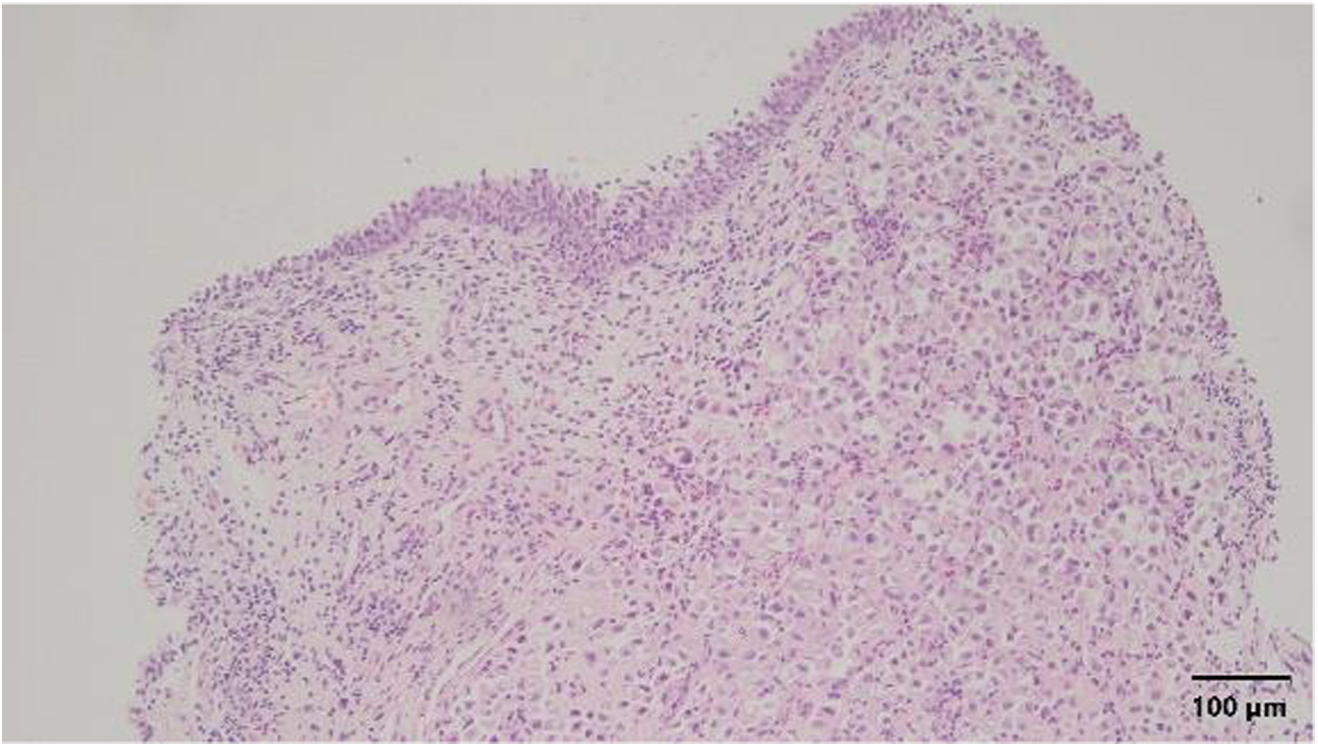
Hematoxylin/eosin‐stained specimen showing tumor cells diffusely infiltrating the submucosa and hyperplastic urothelial cells with mild atypia. Magnification, ×100.

**FIGURE 4:**
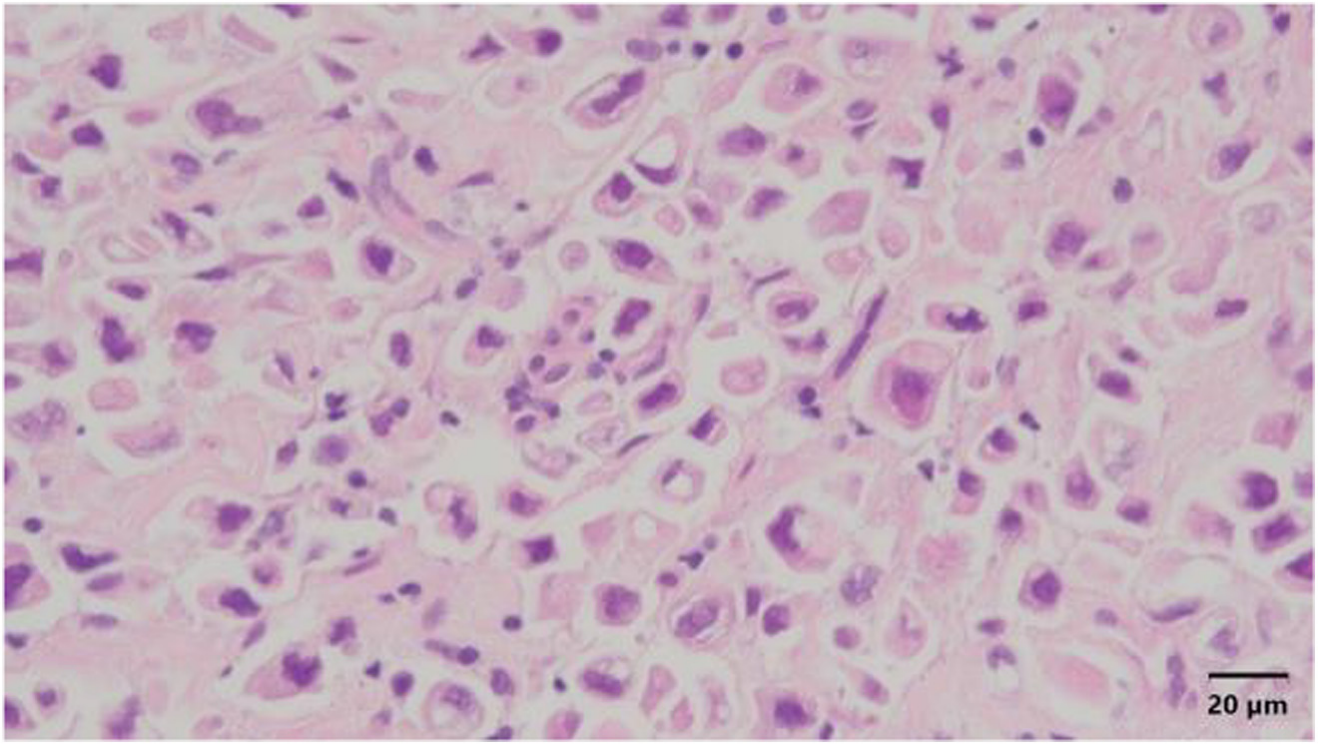
Hematoxylin/eosin‐stained specimen showing tumor cells arranged in a single‐cell pattern with signet‐ring to pleomorphic differentiation. Magnification, ×400.

**FIGURE 5:**
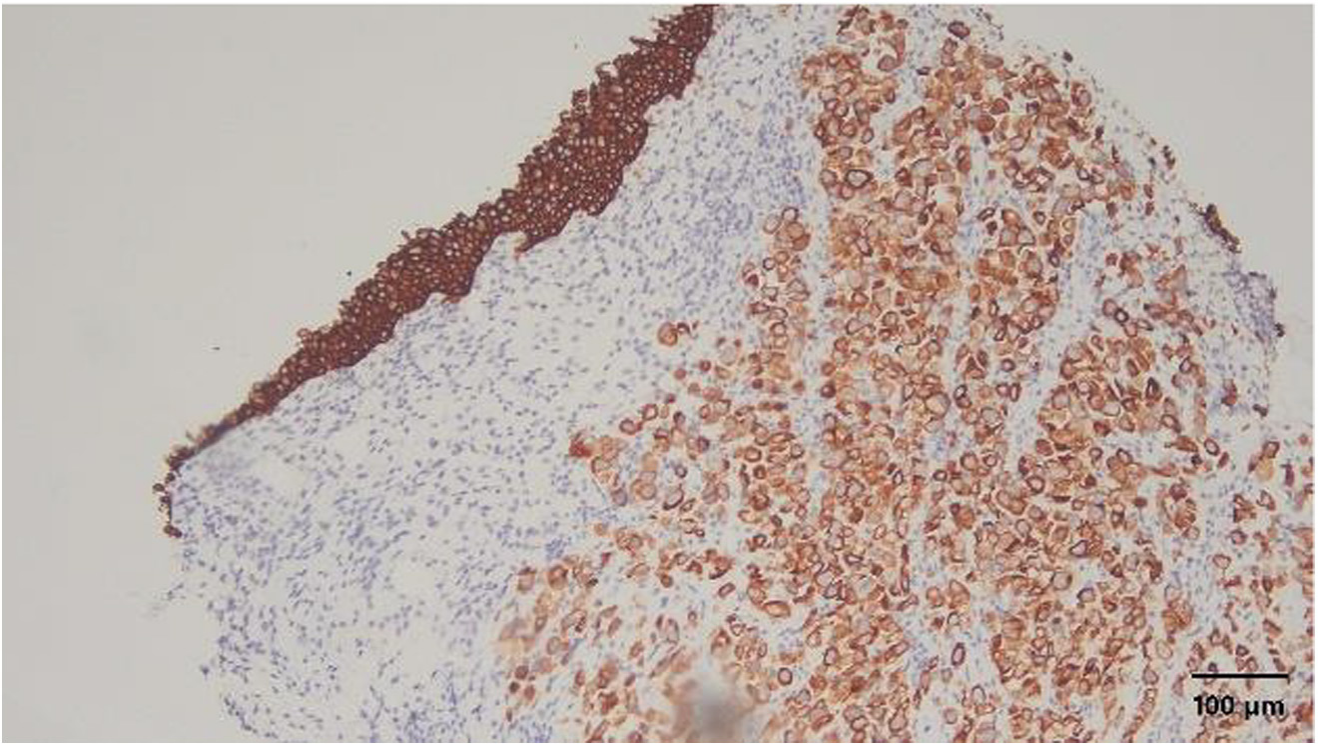
Immunohistochemical staining showing that both the tumor cells and the urothelial mucosa are positive for cytokeratin 34 beta E12. Magnification, ×100.

**FIGURE 6:**
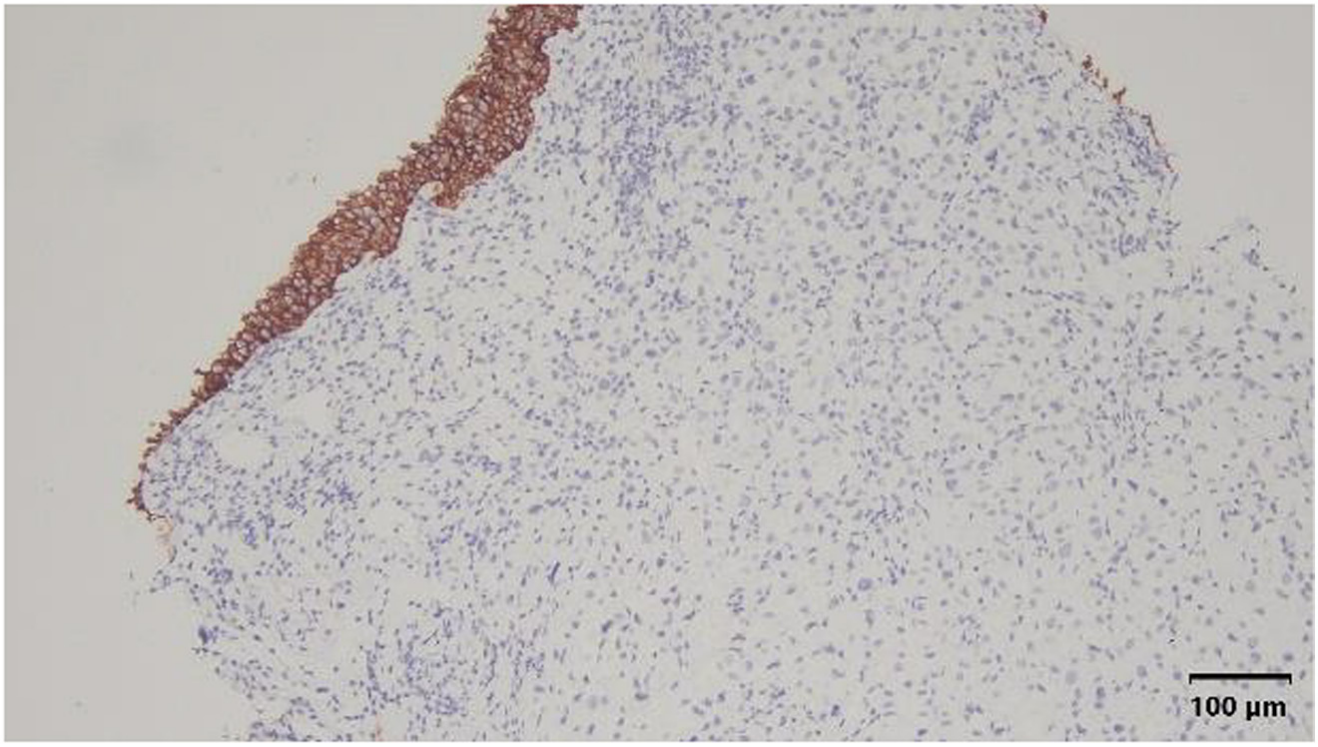
Immunohistochemical staining showing that E‐cadherin is present in urothelial mucosa but not in tumor cells. Magnification, ×100.

Pathological examination of the tumor biopsy specimens revealed metastatic infiltrating lobular carcinoma (ILC) of the signet‐ring cell type. The tumor was poorly differentiated, triple‐negative molecular subtype, and was at high risk of recurrence based on the Immunohistochemical 4 score (Figures 3 and 4). These findings indicated that the tumor was different from the primary papillary carcinoma that was removed 21 years ago. Based on immunostaining, the tumor biopsy specimens were positive for GATA binding protein 3, cytokeratin 34 beta E12 (Figure 5), and Ki67 but negative for epithelial cadherin (E‐cadherin) (Figure 6).

## 5 OUTCOME AND FOLLOW‐UP

After diagnosis, the patient decided to receive further treatment at another hospital due to familial convenience. The family of the patient provided information that the patient received chemotherapy treatment and experienced progressive ascites. Unfortunately, the patient expired from COVID‐19 infection in September, 2022. Further details of her regiment of choice are unavailable owing to the limitations of medical record sharing system.

## 6 DISCUSSION

The second most common cancer in BC survivors is another BC. The new cancer can develop in the same or opposite breast in patients treated with breast‐conserving surgery.^4^ In this study, we have presented a 67‐year‐old female with a history of stage 1 (T1N0M0) papillary carcinoma of the right breast, which was successfully treated by performing modified radical mastectomy (MRM) during initial diagnosis 21 years ago. She was diagnosed with poorly differentiated metastatic ILC with clinical presentation that showed common urinary symptoms. Importantly, the former BC was positive for ER and PR, whereas the current metastatic BC was triple‐negative molecular subtype. Based on the pathology report, the ILC in the bladder might have originated from the left breast. However, we did not conduct any tests for BC, including physical examination, laboratory tests, and imaging, before performing the random biopsy, the results of which indicated metastasis. It was not possible to determine the time of onset for the second BC. It remains possible that the histologic type of the first BC was misdiagnosed due to inadequate pathologic sections or the lack of effective immunostaining markers for diagnostic assistance, since the pathologic diagnosis of ILC is challenging and depends on the quality of the sections.

The differential diagnoses of the initial urinary symptoms to out‐patient‐department including infection, urinary stones, pelvic mass or organ prolapse, tumor or interstitial cystitis. Infection and urinary stones was examined by history, routine urinalysis and X‐ray. The possibility of pelvic organ prolapse was excluded by performing gross inspection and palpation when uroflowmetry was performed. Thus, based on the uroflowmetry results, chronic interstitial cystitis was confirmed without performing further image evaluation. However, her symptoms showed poor response to our treatment, which revealed the chances of the presence of tumor.

Common metastatic sites for BC vary depending on the histologic type. Infiltrating ductal carcinoma (IDC) accounts for approximately 90% of all BCs, followed by ILC (approximately 10%), whereas papillary carcinoma accounts for <1%. Common metastatic sites for IDC are lung, liver, bone, and brain, whereas ILC tends to spread to the gastrointestinal and genitourinary tracts, peritoneum, retroperitoneum, and leptomeninges. ^5^, ^6^, ^7^ Secondary urinary bladder tumors from solid tumors are rare, and most are direct extensions from another pelvic tumor. Metastasis from distant organs are extremely rare; the most common original sites are stomach, lung, and skin (malignant melanoma).^3^ In a literature review of metastatic cancers involving the urinary bladder, Velcheti et al. reported that BC was the primary tumor in approximately 8.5% of the cases,^8^ and only 50 cases has been reported in the last 60 years.^3^, ^8^ The rate of bladder metastasis is higher with ILC than with IDC.^3^

Francesca et al. reviewed hypothesized mechanisms of bladder metastasis were retroperitoneal involvement, hematogenous transport, or lymph node transport.^3^ In comparison with the IDC, E‐cadherin expression is markedly reduced or absent in the great majority of ILCs.^9^ The loss of E‐cadherin, a cell‐to‐cell adhesion molecule, may facilitate metastasis. The mechanism of BC metastasis to the urinary bladder remains poorly understood.

Some patients with metastatic BC in the bladder are asymptomatic, and the tumor is incidentally identified through imaging studies during regular follow‐up. Clinical presentation is diverse, including back pain, hematuria, and voiding dysfunction, such as frequency, urgency, incontinence, and nocturia.^3^ Detrusor involvement may also lead to ureteral obstruction; several cases of bladder metastasis from BC presented with symptomatic hydronephrosis and renal failure.^3^ Comprehensive evaluation with routine urinalysis, urinary culture, and imaging studies such as plain X‐ray of the kidneys, ureters, and bladder and CT can aid in the exclusion of urinary tract infection and urolithiasis. In patients with urinary tract symptoms and a history of malignancy, bladder ultrasound is recommended as a first‐line screening for suspected bladder metastasis. CT is recommended in patients with persistent symptoms and negative ultrasound findings. Magnetic resonance imaging and positron emission tomography/CT are useful in revealing other metastatic sites. In imaging studies, bladder wall thickening is a common finding of bladder metastasis. Urine cytology findings vary and are not sufficiently specific for definite diagnosis.

Elevated levels of CA15‐3, CEA, tissue polypeptide specific antigen, and CA‐125 have been shown to be associated with greater tumor size, lymph node metastasis, and aggressive histology in advanced BC.^10^ Additionally, the combined evaluation of tumor markers improves diagnostic sensitivity compared to single tumor markers.^11^ Therefore, the levels of several tumor markers were evaluated in the current patient; the CA15‐3 level was within the normal range, which could have been misleading in the absence of data on the other tumor markers. Indeed, the elevated serum AFP, CEA, CA‐125, and CA‐199 levels might be an indication of advanced presentation in the current patient.

Definitive diagnosis depends on the pathologic evaluation of tumor biopsy specimens. Transurethral resection of the vesical lesion not only serves as a diagnostic purpose but also ameliorates urinary symptoms and facilitates ureteral stenting to resolve ureteral obstruction.^3^ However, in the present case transurethral resection of the bladder tumor was not suitable due to diffuse thickening of the urinary bladder wall. Therefore, random biopsy specimens of the urinary bladder were collected using cystofibroscopy.

Bladder metastasis from the breast may occur months or years after the initial diagnosis of the primary tumor. The interval between the primary tumor and metastasis can be as long as 30 years, with a mean interval of 90 months.^3^ According to 2012 American Society of Clinical Oncology guidelines for surveillance after BC treatment, all women should undergo physical examination every 3–6 months for the first 3 years after primary therapy, then every 6–12 months for the next 2 years, and then annually. Nevertheless, there was no follow‐up record since her third year after she underwent modified radical mastectomy, which might be the reason for delayed detection of advanced disease progression.

The management of BC metastasis to the urinary bladder includes symptomatic relief and local and systemic therapy. Transurethral resection of the bladder tumor can alleviate tumor burden and facilitate ureteral stenting for ureteral obstruction. Local radiotherapy can stop vesical bleeding and control local disease. The standard therapy includes chemotherapy and hormonal treatment. ER‐positive and PR‐positive tumors are more responsive to hormone therapy than those negative for these receptors and are associated with longer disease‐free survival.^12^ Generally, patients with metastatic BC in the bladder have a very limited survival, ranging 1–24 months. The prognosis is usually poor unless bladder is the only metastatic site.^3^

The association between second primary BCs and urinary bladder metastases is unknown, based on our search of PubMed, Medline, and the Cochrane Library for reviews and case reports using the keywords “second primary BC” AND “urinary bladder metastasis.”

In summary, this case illustrated that the presence of a common symptom could be originated from an uncommon metastatic disease. While the urinary tract is a rare metastatic site for malignancy, especially in BC, clinicians must remain alert in history‐taking despite the ever‐evolving technologies that greatly assist in diagnosis. Taking proper history of the patient can play a critical role in effective diagnosis and reduce the chances of misdiagnosis and wrong treatment. In patients with a history of cancer, the focus should not only be on the currently affected site but also on the status of the underlying malignancy. Certainly, a comprehensive physical examination is needed as well.

## AUTHOR CONTRIBUTIONS

**Chang‐Chieh Liao:** Conceptualization; data curation; formal analysis; writing – original draft. **Yen‐Chuan Ou:** Supervision. **Min‐Che Tung:** Supervision. **Wei‐Shiang Hu:** Investigation. **Tang‐Yi Tsao:** Investigation. **Chao‐Yu Hsu:** Project administration; supervision; writing – review and editing.

## FUNDING INFORMATION

This report did not receive any specific grants from funding agencies in the public, commercial, or not‐for‐profit sectors.

## CONFLICT OF INTEREST STATEMENT

The authors declare that they have no competing interests.

## PATIENT CONSENT

Written informed consent was obtained from the patient's daughter due to the patient has expired.

## Data Availability

Not applicable.
